# R-wave synchronised atrial pacing in pediatric patients with postoperative junctional ectopic tachycardia: the atrioventricular interval investigated by computational analysis and clinical evaluation

**DOI:** 10.1186/s12938-017-0430-z

**Published:** 2017-12-19

**Authors:** Andreas Entenmann, Miriam Michel, Bruno Ismer, Roman Gebauer

**Affiliations:** 1 Department of Pediatrics, Innsbruck Medical University, Anichstrasse 35, 6020 Innsbruck, Austria; 2 Department for Congenital Heart Disease and Pediatric Cardiology, Schleswig–Holstein University Hospital, Arnold-Heller-Strasse 3, 24105 Kiel, Germany; 3 Peter Osypka Institute for Pacing and Ablation, Offenburg University of Applied Sciences, Offenburg, Germany; 4 Department of Pediatric Cardiology, University of Leipzig, Strümpellstrasse 39, 04289 Leipzig, Germany

**Keywords:** Congenital heart defect, Cardiac surgical procedures, Arrhythmia, Junctional ectopic tachycardia, Cardiac pacing

## Abstract

**Background:**

R-wave synchronised atrial pacing is an effective temporary pacing therapy in infants with postoperative junctional ectopic tachycardia. In the technique currently used, adverse short or long intervals between atrial pacing and ventricular sensing (AP–VS) may be observed during routine clinical practice.

**Objectives:**

The aim of the study was to analyse outcomes of R-wave synchronised atrial pacing and the relationship between maximum tracking rates and AP–VS intervals.

**Methods:**

Calculated AP–VS intervals were compared with those predicted by experienced pediatric cardiologist.

**Results:**

A maximum tracking rate (MTR) set 10 bpm higher than the heart rate (HR) may result in undesirable short AP–VS intervals (minimum 83 ms). A MTR set 20 bpm above the HR is the hemodynamically better choice (minimum 96 ms). Effects of either setting on the AP–VS interval could not be predicted by experienced observers. In our newly proposed technique the AP–VS interval approaches 95 ms for HR > 210 bpm and 130 ms for HR < 130 bpm. The progression is linear and decreases strictly (− 0.4 ms/bpm) between the two extreme levels.

**Conclusions:**

Adjusting the AP–VS interval in the currently used technique is complex and may imply unfavorable pacemaker settings. A new pacemaker design is advisable to allow direct control of the AP–VS interval.

## Background

Junctional ectopic tachycardia (JET) is a serious heart rhythm disturbance affecting 1.4–8.0% of all infants and children undergoing surgery for a congenital heart defect [1–3]. The absence of synchronised atrial activity in combination with postoperative ventricular dysfunction may significantly decrease cardiac output and is associated with increased morbidity and mortality [4–6]. Effective therapy is based on administration of antiarrhythmic drugs, deep sedation, and mild hypothermia [7–9]. Different temporary pacing techniques are used, aiming either to restore atrioventricular synchronism or to reduce heart rate [10–12].

**Fig. 1:**
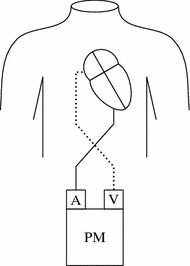
R-wave synchronised atrial pacing by inverse connection of the pacing wires on an external dual chamber pacemaker. *PM* pacemaker, *A* atrial channel, *V* ventricular channel. With friendly permission [12, 15]

In 1991, Till and Rowland described an innovative temporary pacing technique [13]. Their idea was to use a sensed R-wave of junctional tachycardia to serve as a trigger for a paced atrial contraction before the following QRS-complex. According to the North American Society of Pacing and Electrophysiology generic pacemaker code, the method can be described as AVT-pacing, with the first digit encoding the side of pacing (A: atria), the second the site of sensing (V: ventricles) and the third the pacing mode (T: triggered) [4]. Fourteen years later, in 2003, Janoušek et al. introduced AVT pacing by inverse connection of the pacing wires on a commercial external dual chamber pacemaker [14]. Thus, sensing the ventricular action on atrial input, subsequent atrial pacing can be performed via the ventricular output of the pacemaker (Fig. 1).

This approach was successfully used in the treatment of postoperative JET and has become a standard therapy in many centers [2]. Nevertheless, with this technique, individual adjustment of the interval between atrial pacing and ventricular sensing (AP–VS) is demanding. Very short AP–VS intervals may impair hemodynamics and can cause atrial contractions against closed atrioventricular valves. Abnormally long AP–VS intervals may promote the occurrence of pacemaker-induced tachycardia [14]. Our experience has included frequent instances of such adverse events, with a distinct short or long AP–VS interval: Indeed, in one out of ten patients treated with this technique, pacemaker-induced tachycardia occurred. The aim of this study was to analyse the mode of adjusting the AP–VS interval based on the method by Janoušek et al. Our findings lead us to suggest that alternative strategies of adjusting the AP–VS interval should be developed for future pacemaker designs.

## Methods

**Fig. 2:**
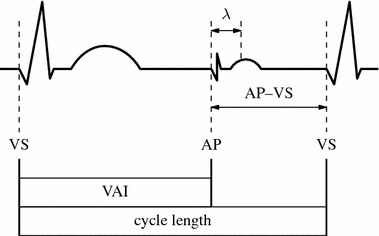
Intervals in AVT pacing. The interval between atrial pacing *(AP)* and ventricular sensing (*VS)* is the difference between the tachycardia’s cycle length and the ventriculoatrial interval *(VAI)* adjusted at the pacemaker. VAI corresponds to the pacemaker’s atrioventricular delay (AV-delay) due to the exchanged pacing wires; *λ* pace-sense offset

In the technique described by Janoušek et al., the AP–VS interval is indirectly adjusted via the maximum tracking rate (MTR). The recommended MTR is reported to be between 10 and 20 bpm above the patient’s tachycardia rate. With respect to these recommended settings we specified two different methods, namely M10 and M20, where the MTR is set 10 bpm and 20 bpm above the tachycardia rate, respectively. Values for the maximum allowed duration of the pacemaker’s AV-delay (effective ventriculoatrial interval) according to the chosen MTR were taken from the article of Janoušek et al. and are displayed in Table 1 [14]. In this context, the primary role of the MTR is not to limit tracking but to serve as a reference rate to determine the maximum duration of the ventriculoatrial interval (Fig. 2).

**Table 1:** Maximum duration of the effective ventriculoatrial interval in the method of Janoušek et al. [14]

MTR (bpm)	VAI_max_ (ms)	MTR (bpm)	VAI_max_ (ms)	MTR (bpm)	VAI_max_ (ms)
230	190	180	260	130	390
220	200	170	280	120	400
210	210	160	300	110	400
200	220	150	320	100	400
190	240	140	350	90	400

*MTR* maximum tracking rate, *VAI*_*max*_ maximum effective ventriculoatrial interval

Thus AVT pacing was performed by setting the MTR 10 or 20 bpm above tachycardia rate and selecting the maximum allowed pacemaker’s AV-delay (effective ventriculoatrial interval, VAI), followed by fine tuning according to the hemodynamic demands of the patient.

The method as a whole is based on the use of a modified external pacemaker (PACE 203H, version JJ or higher, Osypka Medical, La Jolla, CA). The following settings are required: a postventricular atrial refractory period (PVARP) of 100 ms, ventricular sensing set limitlessly high, and atrial sensing adjusted to half of the measured input signal. Due to the exchange of the pacing wires, the PVARP thus effectively serves as postatrial ventricular refractory period (PAVRP)—which should help avoid misinterpretation of effective atrial stimulation as ventricular excitation, and the pacemaker is now blinded for signals from the atrium and is triggered by ventricular excitations via the atrial channel. Maximal tracking rates can be set in a range from 80 to 230 ppm, allowing AVT pacing in patients with junctional heart rates of up to 220 ppm.

### Computational analysis

In order to analyze which settings yield the best results for the duration of the AP–VS interval using the technique described by Janoušek et al., we calculated the respective durations for the two different maximum tracking rates, MTR_10_ (method M10) and MTR_20_ (method M20). The AP–VS interval was calculated as difference between the tachycardia’s cycle length and the ventriculoatrial interval. According to the MTR used, the values for the maximum duration of the ventriculoatrial interval (VAI_max_) were extracted from Table 1 as proposed by Janoušek et al. In the following, AP–VS_10_ signifies the AP–VS interval resulting from a MTR set 10 bpm higher than the patient’s tachycardia rate and a VAI set to its maximum allowed duration (method M10). Similarly, AP–VS_20_ stands for the interval resulting from a MTR set 20 bpm higher than the junctional heart rate with the respective VAI_max_ (method M20).

In order to illustrate the impact of both methods, M10 and M20, we plotted the intervals AP–VS_10_ and AP–VS_20_ versus the tachycardia rate in comparison to the course of normal PQ duration plus 0, 10, and 20 ms. Therefore, heart rate-related PQ durations were derived from the literature [16, 17].

#### Clinical evaluation

To test the feasibility of the two methods, we evaluated whether pediatric cardiologists can accurately assess the effects of the M10 and M20 approach on the resulting AP–VS interval in a standardized testing environment [15]. Therefore, junctional tachycardia rates ranging from 100 to 220 bpm were presented to six fully trained pediatric cardiologists with solid expertise in AVT pacing. They were asked to state how long they expected the AP–VS intervals to last using either method, M10 or M20. The resulting estimated AP–VS intervals were compared with the calculated intervals.

#### Intervention thresholds

In application of AVT pacing, the tachycardia rate of the patient frequently changes in the course of the disorder. This requires readjustment of the pacemaker settings in order to avoid adverse interval durations. In accord with our clinical experience, intervention thresholds were defined by AP–VS intervals between 80 and 165 ms, which were proven to be safe in order to avoid simultaneous contraction of atria and ventricles or pacemaker induced tachycardia. Provided that the VAI is not changed and remains maximal according to the original heart rate, the AP–VS interval can be calculated for heart rates and cycle lengths above and below the original heart rate.

#### Rate-related adjustment

As a prerequisite for an automated rate-related AP–VS adjustment, we calculated and defined an idealised relationship between AP–VS interval and tachycardia rate. All underlying assumptions were based on data published by Ritter et al., Ismer et al. and Koglek et al. [18–20]. A formula was developed based on the work of Butterworth and Bode to approximate the operation characteristics [21, 22].

#### Statistics

All analyses were done using the statistical software SPSS 20.0 (SPSS, Chicago, IL) and Microsoft Office Excel 2007 (Microsoft, Redmond, WA). The strength and direction of linear relationship were expressed as correlation coefficients R^2^ by Pearson.

For calculations in the context of the newly developed formula we used the free interpreted programming language, Perl, by Larry Wall [23].

## Results

### Computational analysis

**Fig. 3:**
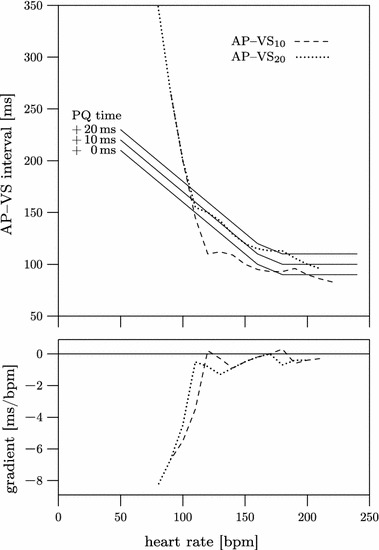
Calculated intervals between atrial pacing and ventricular sensing *(AP*–*VS)* vs. heart rate with normal PQ durations based on literature as a reference (top). Graphs of normal PQ durations + 10 ms and + 20 ms were plotted to facilitate visual perception of changes in time lengths. Gradients of both calculated AP–VS curves (bottom)

The characteristic difference between method M10 and M20 became evident when the calculated values of the AP–VS_10_ and AP–VS_20_ intervals were plotted against the heart rate values of the normal PQ duration plus 0, 10, and 20 ms (Fig. 3).

The AP–VS_10_ graph has a tendency to generate very short AP–VS intervals, with a minimum duration of 83 ms. The curve is not linear. Gradient values higher than zero indicate that the graph does not monotonically decrease with increasing heart rates. Monotonically increasing or decreasing in this context means, that the graph is strictly increasing or decreasing, therefore its gradient is always positive (increasing graph) or always negative (decreasing graph) and never zero. The AP–VS_20_ curve, in contrast, fits almost gently to the reference curve of the normal PQ interval plus 10 ms. The minimum duration of the AP–VS_20_ interval is 96 ms for very high frequencies. The curve is monotonic-decreasing as the overall gradient is ≤ 0. For heart rates less than 120 bpm (AP–VS_10_) or 110 bpm (AP–VS_20_), both graphs steeply slope downwards with increasing tachycardia rates. This is due to the fixed VAI_max_ of 400 ms for maximum tracking rates below 130 bpm (Table 1). Considering this fact and in order to avoid falsifications we analyzed regression lines only for heart rates ranging from 130 to 220 bpm. As AVT pacing usually is performed at heart rates above 130 bpm this does not constitute a restriction. The correlation coefficients R^2^ between the calculated values of the AP–VS graphs and their deduced regression lines were 0.87 and 0.94 for AP–VS_10_ and AP–VS_20_, respectively, signalling that the AP–VS_20_ graph is closer to perfect linearity than is the AP–VS_10_ graph.

#### Clinical evaluation

**Fig. 4:**
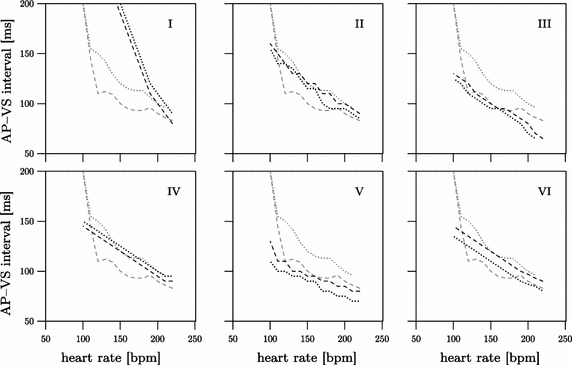
AP–VS_10_ (dashed line) and AP–VS_20_ intervals (dotted line) anticipated by six experienced observers (I–VI). Fine lines in the background indicate the calculated AP–VS intervals as a reference. *AP* atrial pacing, *VS* ventricular sensing

When six pediatric cardiologists were asked to estimate the resulting AP–VS intervals by setting the MTR either 10 or 20 bpm above the given heart rate and selecting the effective VAI to the maximum allowed duration, it became evident that all observers expected an almost linear and monotonic-decreasing relationship (Fig. 4).

Analysis of the regression lines derived from the estimated values revealed that all observers expected a more linear relationship between AP–VS interval and tachycardia rate than that which the calculated AP–VS graphs provide (Table 2).

**Table 2:** Characteristics of the regression lines from AP–VS intervals calculated or estimated by six observers (I–VI)

	Calculated		I		II		III		IV		V		VI	
AP–VS_10/20_	10	20	10	20	10	20	10	20	10	20	10	20	10	20
Gradient	− 0.28	− 0.56	− 2.06	− 2.06	− 0.49	− 0.58	− 0.48	− 0.48	− 0.50	− 0.50	− 0.29	− 0.33	− 0.48	− 0.46
Intercept	143	209	506	516	196	206	174	168	195	200	140	140	191	179
R^2^	0.87	0.94	0.97	0.97	0.97	0.93	0.97	0.97	1.0	1.0	0.94	0.97	0.99	0.99

Gradient in ms/bpm, intercept in ms

Four of six observers (II, III, V, VI) erroneously believed the AP–VS_10_ values to be higher than AP–VS_20_ values. Noticeably, all observers assumed that the two AP–VS graphs have an almost parallel course. In fact, graphs of the calculated AP–VS intervals do not run parallel. Five observers (II, III, IV, V, VI) expected a gradient close to the calculated AP–VS_20_ graph. Only one observer (I) suspected a slope clearly steeper than the calculated AP–VS_10_ or AP–VS_20_ gradient. To sum up, the calculated AP–VS intervals differ markedly from the estimated intervals and therefore cannot be assessed by intuition.

#### Intervention thresholds

Effects of increasing or decreasing heart rates on the resulting AP–VS interval are illustrated in Table 3.

**Table 3:** Effects of a changing tachycardia rate on the AP–VS interval

HR_0_	∆HR									
	± 0 bpm		+ 5 bpm		+ 10 bpm		− 10 bpm		− 20 bpm	
	AP–VS		AP–VS		AP–VS		AP–VS		AP–VS	
	10	20	10	20	10	20	10	20	10	20
220	83		77		71		96		110	
210	86	96	79	89	73	83	100	110	116	126
200	90	100	83	93	76	86	106	116	123	133
190	96	10	88	98	80	90	113	123	133	143
180	93	113	84	104	76	96	113	133	135	155
170	93	113	83	103	73	93	115	135	140	160
160	95	115	84	104	73	93	120	140	149	169
150	100	120	87	107	75	95	129	149	162	182
140	109	129	94	114	80	100	142	162	180	200
130	112	142	94	124	79	109	150	180	195	225
120	110	150	90	130	72	112	155	195	210	250
110	145	155	122	132	100	110	200	210	267	277
100	200	200	171	171	145	145	267	267	350	350
90	267	267	232	22	200	200	350	350	457	457

*HR*_*0*_ original heart rate, *∆HR* change in heart rate, *AP* atrial pacing, *VS* ventricular sensing

For high and increasing tachycardia rates method M20 is more suitable than method M10. Aiming to avoid AP–VS durations < 80 ms no further increase in heart rate more than 5 bpm is sought to be tolerable using method M10 at tachycardia rates > 200 bpm. In comparison, the threshold for readjusting the effective VAI is 10 bpm above the original heart rate (HR_0_) for method M20 in this situation. For decreasing heart rates, in contrast, long AP–VS intervals (≥ 165 ms) are rather a problem with method M20 than with method M10. Using method M20, decreases in heart rate of more than 20 bpm have to be addressed by readjustments of the VAI if the original heart rate H_0_ is ≥ 170 bpm. If H_0_ is < 170 bpm, a decrease of only 10 bpm can be tolerated.

#### Rate-related adjustment

In case of retrograde conduction from the junctional ectopic focus to the atria the duration of the AP–VS interval has to be minimized for high tachycardia rates. This will avoid atrial pacing during the atrial refractory period started by spontaneous retrograde atrial activation. AP–VS intervals may subsequently be fine-tuned once 1:1 retrograde conduction from the junctional focus is interrupted. On the other hand, the duration of the AP–VS interval in relation to the tachycardia rate is also limited for descending heart rates to avoid pacemaker-induced tachycardia.

**Fig. 5:**
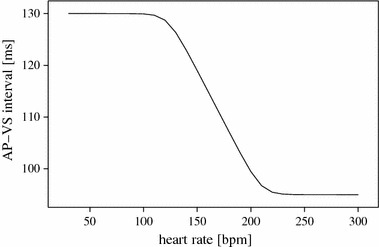
Proposed relationship between junctional heart rate and ideal AP–VS interval

The “ideal AP–VS interval” with regard to hemodynamics allows enough time for passive early diastolic filling of the atria and for active atrial contraction. The physiologic behavior of the atrioventricular node therefore is to lengthen the PQ interval with decreasing heart rates (if more time is available) and vice versa with increasing heart rates. This heart rate related change in PQ duration is about 0.4 ms/bpm [25]. In AVT pacing the time between atrial pacemaker stimulation and atrial myocardial contraction (the time necessary for an atrial stimulus to propagate throughout the atrial myocardium) has to be considered (pace-sense offset). Including the pace-sense offset we calculated the ideal AP–VS interval as between 95 ms for high tachycardia rates and 130 ms for low heart rates (Fig. 5).

The method works with an effective PAVRP duration of 90 ms to allow a sensing window of at least 5 ms even for very high tachycardia rates. The progression between the two extreme levels of the AP–VS interval was chosen to be linear and strictly monotonic-decreasing with respect to the AV node’s biological behavior (− 0.4 ms/bpm).

The relationship between the AP–VS interval and the junctional heart rate (HR) as shown in Fig. 5 is approximated by the mathematical function *y* = *f(x)*, where *x* = HR (bpm)/100 bpm and *y* = AP–VS (ms)/100 ms.

1a $$\begin{aligned} y(x) & = \frac{1}{2}\frac{m}{n}\mathop \sum \limits_{{i = 1}}^{{n/2}} \left\{ {\ln \left[ {\left( {1 - e^{{2(x - x_{2} )}} } \right)^{2} + \frac{{e^{{2(x - x_{2} )}} }}{{Q_{i}^{2} }}} \right]} \right. \\ & \left. { \quad - \;\ln \left[ {\left( {1 - e^{{2(x - x_{1} )}} } \right)^{2} + \frac{{e^{{2(x - x_{1} )}} }}{{Q_{i}^{2} }}} \right]} \right\} + c \end{aligned}$$

1b $$Q_{i} = \frac{1}{{2{\text{sin}}\phi _{i} }}, \quad \;\phi _{i} = \frac{\pi }{{2n}}\left( {2i - 1} \right), \quad \;i = 1, \ldots ,\frac{n}{2}.$$

With the following parameter settings, *x*_1_ = 1.225, *x*_2_ = 2.1, *m* = 0.4 (slope), *c* = 1.3 (AP–VS_max_), *n* = 8, we obtained the curve shown in Fig. 5. The minimum value of AP–VS is then given by *c* − *m(x*_2_ − *x*_1_) as 95 ms.

## Discussion

Our study demonstrates the complexity of AVT pacing by indirectly adjusting the AP–VS interval via the MTR as proposed by Janoušek et al. [14]. Selecting the M20 method with MTR 20 bpm above the junctional tachycardia rate proved the best approach when setting the effective VAI to its maximum allowable duration. The attained AP–VS_20_ intervals then approximate the duration of a normal PQ interval plus 10 ms. This seems to be hemodynamically useful as it allows the atrial pacing stimulus to propagate throughout the atrial myocardium, as proposed by Ausubel et al. [24]. For high junctional rates the resulting minimal AP–VS_20_ interval is 96 ms, which is an adequate interval in this special situation. The method is limited to junctional rates ≤ 210 bpm, as the maximum adjustable MTR is 230 bpm. Analysing method M10, the AP–VS_10_ curve runs at a relatively even level and involves very short AP–VS values. The shape of the AP–VS_10_ curve is not properly anticipated even by experienced observers.

The reason for both the exceptional shape of the graph and the errors in intuitive assessment is the nonlinear (hyperbolic) relationship between heart rate and cycle length. Therefore, the resulting AP–VS intervals were unforeseen by all pediatric cardiologists in our experiment. As a consequence, ECG recordings should be done with every readjustment of the pacemaker, especially if the selected MTR value is less than 20 bpm above the junctional heart rate, in order to avoid unintended short AP–VS intervals.

In summary, method M20 is superior to method M10 in preventing short AP–VS intervals at high heart rates. On the other hand, there is a higher risk for adverse long AP–VS intervals with decreasing heart rates using method M20. Thus, pacemaker-induced tachycardia may occur if the AP–VS interval is long enough to allow antegrade atrioventricular conduction of the atrial pacing stimulus as described by Janoušek et al. [14]. The steeply descending graphs of both calculated AP–VS intervals at low junctional rates illustrate the risk of adverse long AP–VS intervals with decreasing heart rates (Fig. 3). These highly negative gradients result from a VAI_max_ fixed to 400 ms for maximum tracking rates less than 130 bpm. We consider this technical implementation as a safety risk of the method by Janoušek et al. and favor the idea of direct AP–VS adjustment in future pacemaker designs. An automated, direct and tachycardia rate-related technique for AP–VS adjustment would reduce the need for frequent readjustments and close monitoring during the course of AVT pacing. The basis of this approach requires a statement about the ideal relationship between AP–VS interval and heart rate. The AP–VS interval consists of the AV interval that is hemodynamically most beneficial and the pace-sense offset. Information about both is published [18–20, 24]. Between its upper and lower limitations in duration the AP–VS interval in our model mimics the biological behavior of the AV node according to Davignon [25]. Thus, as a next step, based on these findings, an automatic algorithm to calculate any AP–VS duration with respect to the patient’s tachycardia rate should be developed for future external pacemakers.

Although an automatic AP–VS algorithm will make AVT pacing more comfortable to use and increase safety and effectivity of the method, however, the calculated interval may not inevitably be the hemodynamically best AP–VS interval in every patient at a particular time during therapy. The ideal length of the interval depends on many individual factors such as the extent of diastolic and systolic dysfunction, conduction delays within the atrial myocardium, or the localization of atrial or ventricular pacing wires. Therefore, another feature of a new pacemaker design should be the possibility to adjust relative changes to the calculated AP–VS interval (e.g. calculated interval + 10 ms). Furthermore, in some cases, any automatic extent of the AP–VS interval with decreasing junctional heart rates will interfere with effective AVT pacing, e.g., when 1:1 retrograde conduction of the ventricular stimulus to the atria generates an atrial refractory period and allows only a very short interval for atrial activation. Therefore an AVT-capable pacemaker has to provide also the feature of non-automatic direct AP–VS interval adjustment in combination with a warning message if intervals are chosen beyond the safety limits of < 80 or > 165 ms.

### Limitations

Experimental data concerning the hemodynamically ideal AP–VS interval during JET in young infants are not available. The relationship between AP–VS interval and junctional rate stated in this paper is deduced from clinical experience and theoretical considerations. Another source of data is studies of conventional pacing techniques in adult patients. These data may not fit the needs of an infant suffering from postoperative JET with very high heart rates and severely compromised systolic and diastolic cardial function. As the number of patients with postoperative JET is limited, prospective multicenter studies are necessary to investigate the hemodynamically optimized AP–VS interval during AVT-pacing.

## Conclusions

This study describes the technical details of AVT pacing and provides information about advantageous and disadvantageous pacemaker settings. Our study leads us to recommend a re-design of an AVT-capable external pacemaker to make the method safer, more effective, and easier to use. The new device should provide a distinct AVT mode featuring an internal exchange of the atrial and ventricular channel. AP–VS intervals should be directly adjusted by the user. Automatic rate-related adjustment of the AP–VS interval can be achieved depending on internally measured cycle lengths. The proposed relationship between AP–VS intervals and junctional heart rate is suitable in the context of this study until proven otherwise by data from further studies.

## Abbreviations

AP: atrial pacing
AV: atrioventricular
AVT pacing: R-wave synchronized atrial pacing
HR: heart rate
JET: junctional ectopic tachycardia
PAVRP: postatrial ventricular refractory period
PVARP: postventricular atrial refractory period
MTR: maximum tracking rate
R^2^: correlation coefficient by Pearson
VA: ventriculoatrial
VAI: ventriculoatrial interval
VS: ventricular sensing
